# Agricultural practices altered soybean seed protein, oil, fatty acids, sugars, and minerals in the Midsouth USA

**DOI:** 10.3389/fpls.2015.00031

**Published:** 2015-02-18

**Authors:** Nacer Bellaloui, H. Arnold Bruns, Hamed K. Abbas, Alemu Mengistu, Daniel K. Fisher, Krishna N. Reddy

**Affiliations:** ^1^Crop Genetics Research Unit, Plant Physiology, United States Department of Agriculture – Agricultural Research ServiceStoneville, MS, USA; ^2^Crop Production Systems Research Unit, Crop Production, United States Department of Agriculture – Agricultural Research ServiceStoneville, MS, USA; ^3^Biological Control of Pests Research Unit, Biological Control, United States Department of Agriculture – Agricultural Research ServiceStoneville, MS, USA; ^4^Crop Genetics Research Unit, Pathology, United States Department of Agriculture – Agricultural Research ServiceJackson, TN, USA

**Keywords:** seed protein, fatty acids, sugars, minerals, twin-rows, seeding rate, planting date

## Abstract

Information on the effects of management practices on soybean seed composition is scarce. Therefore, the objective of this research was to investigate the effects of planting date (PD) and seeding rate (SR) on seed composition (protein, oil, fatty acids, and sugars) and seed minerals (B, P, and Fe) in soybean grown in two row-types (RTs) on the Mississippi Delta region of the Midsouth USA. Two field experiments were conducted in 2009 and 2010 on Sharkey clay and Beulah fine sandy loam soil at Stoneville, MS, USA, under irrigated conditions. Soybean were grown in 102 cm single-rows and 25 cm twin-rows in 102 cm centers at SRs of 20, 30, 40, and 50 seeds m^-2^. The results showed that in May and June planting, protein, glucose, P, and B concentrations increased with increased SR, but at the highest SRs (40 and 50 seeds m^-2^), the concentrations remained constant or declined. Palmitic, stearic, and linoleic acid concentrations were the least responsive to SR increases. Early planting resulted in higher oil, oleic acid, sucrose, B, and P on both single and twin-rows. Late planting resulted in higher protein and linolenic acid, but lower oleic acid and oil concentrations. The changes in seed constituents could be due to changes in environmental factors (drought and temperature), and nutrient accumulation in seeds and leaves. The increase of stachyose sugar in 2010 may be due to a drier year and high temperature in 2010 compared to 2009; suggesting the possible role of stachyose as an environmental stress compound. Our research demonstrated that PD, SR, and RT altered some seed constituents, but the level of alteration in each year dependent on environmental factors such as drought and temperature. This information benefits growers and breeders for considering agronomic practices to select for soybean seed nutritional qualities under drought and high heat conditions.

## INTRODUCTION

Soybean is a major oil and protein crop globally. The seed quality is determined by its composition, including protein, oil, fatty acids, sugars, and minerals. Soybean seed contains on a dry weight basis about 380 to 420 g kg^-1^ protein, 190 to 230 g kg^-1^ oil, and based on the total oil, 120 to 130 g kg^-1^ palmitic acid, 30 to 40 g kg^-1^ oleic acid, 480 to 580 g kg^-1^ linoleic acid, and 50 to 80 g kg^-1^ linolenic acid. They also contain sugars such as monosaccharides (glucose and fructose), disaccharide (sucrose), and oligosaccharides (raffinose and stachyose). The mineral composition includes P, K, Ca, Mn, Zn, Fe, and B that are essential for human nutrition, and deficiency of these minerals in the diet can lead to human malnutrition and health problems (Samman et al., 1998; Bouis, 2003; Devirian and Volpe, 2003; Lu et al., 2008). It was reported that over 3 billion people are suffering from malnutrition of minerals, especially iron and zinc (Welch and Graham, 2004; Lu et al., 2008; White and Broadley, 2009). Higher oleic, and lower linoleic and linolenic acids are desirable because they contribute to oil stability. Lower raffinose and stachyose and higher sucrose, fructose, and glucose are desirable because mono- and disaccharides contribute to flavor and taste, but high raffinose and stachyose are indigestible and cause flatulence and diarrhea in non-ruminant animals (Liu, 1997). Seed composition constituents are genetically controlled; however, they are known to be influenced by biotic and abiotic factors such as genotype, maturity, growing season, geographic location, and agronomic practices (Harue and Hirokadzu, 1971; Chapman et al., 1976; Chy and Sheldon, 1979; Wilcox and Cavins, 1995).

Traditionally soybeans in the Midsouth are grown in single-rows, and between row-spacings of 88 to 102 cm are common for soybeans produced in the Mississippi Delta (Ebelhar, 2010). With the commercialization and availability of twin-row-planters (Mascagni et al., 2008; Bruns, 2011), more farmers in the Mississippi delta are becoming interested in twin-row production. Twin-row planters have the capability of planting twin-rows 7.5 to 10 inches (19.1 to 25.4 cm) apart. Although yield responses of single-row vs. twin-row planting have been inconsistent across crops, years, and locations, soybean still has the largest positive response to yield increase in twin-rows vs. single-row (Mascagni et al., 2008). Also, in the Midsouth, including Mississippi, some growers plant soybean as a double-crop with wheat (Lehrsch et al., 1994; Minor and Wiebold, 1998; Heatherly, 2014), and in this production system, soybeans are usually planted in June. This is considered late planting, as opposed to early planting which usually occurs in April. Late planting exposes soybean to a new environment of drought, heat, photoperiod, and diseases, especially charcoal rot and phomopsis. In the Early Soybean Production System (ESPS) in the Midsouth, soybean cultivars of maturity group (MG) IV and V are planted in April–May and harvested in August–September (Heatherly, 1999; Ray et al., 2006) to avoid drought stress during late July through early September. In spite of the yield benefit of the ESPS (Heatherly et al., 1999; Ray et al., 2006), poor seed quality (Mengistu and Heatherly, 2006; Mengistu et al., 2007; Smith et al., 2008), and variability of seed constituents (Bellaloui et al., 2008, 2009b) remain a challenge. Therefore, optimizing the ESPS in the Midsouth for higher, sustainable seed quality is critical.

Although limited information is available on the effects of planting date (PD; Schnebly and Fehr, 1993; Jaureguy et al., 2013) and row-spacing (Boydak et al., 2002), and seeding rate (SR) and row-spacing (Bellaloui et al., 2014) on seed composition, to our knowledge there has been no research done on the combined effects of PD, row-type (RT), and SR on seed composition and mineral nutrition. Therefore, the objective of the current research was to evaluate the effects of single- and twin-row plantings (using 102 and 25 cm on 102 cm center row-spacing, respectively), early, intermediate, and late planting, and SRs of 20, 30, 40, and 50 seeds m^-2^ on soybean seed composition in sandy and clay soils under the Midsouth agro-ecosystem. Our hypothesis was that PD combined with RT (single- vs. twin) and SR will subject the crop to a new growing environment, altering seed composition constituents.

## MATERIALS AND METHODS

### FIELD MANAGEMENT AND GROWTH CONDITIONS

An experiment was performed under field conditions in 2009 and 2010 on Sharkey clay (very-fine, smectitic, thermic Chromic Epiaquerts) and sandy loam (Beulah fine sandy loam, coarse-loamy, mixed, active, thermic Typic Dystrudepts) soils in Stoneville, MS, USA. The current research focused on seed nutrition only, and the agronomic component, including yield, was previously published (Bruns, 2011). Field management and growth conditions were described in detail previously (Bruns, 2011). Briefly, single-row plots were planted using an Almaco cone plot planter (Allen Machine Company, Nevada, IA, USA), and twin-row plots were planted using a four unit Monosem NG-3 (Monosem, Edwardsville, KS, USA) twin-row planter set on 102-cm centers and 25 cm between rows. In the Sharkey clay soil, soybean was planted in 2009 on 22 April, 20 May, and 17 June. In 2010, plantings occurred on 12 April, 11 May, and 2 June. In the sandy loam soil, PDs were 8 April, 11 May, and 8 June in 2009, and 14 April, 11 May, and 17 June in 2010. Cultivar Pioneer brand, 94B73, representative of most of the cultivars grown in the ESPS in the Midsouth was used. For weed control, a pre-plant application of trifluralin [2,6-dinitro-*N*,*N*-dipropyl-4-(trifluoromethyl)aniline] at 0.7 kg ai ha^-1^ was applied, followed by two post-emergence applications of metolachlor [2-chloro-*N*-(2-ethyl-6-methylphenyl)-*N*-(2-methoxy- 1-methylethyl) acetamide] and glyphosate [2-[(phosphonomethyl)amino]acetic acid] at growth stage V2 to V3 (two to three trifoliolates) and at V5 to V6 (five to six trifoliolates). To control fungus, pyraclostrobin (carbamic acid, [[[[1-(4-cholrophenyl)-H-pyrazol-3-yl]oxy]methyl]phenyl]methoxy-,methyl ester) was applied at factory label directions at V5 to V6. The experiments were furrow irrigated starting at R1 (beginning flowering) through R6 (full seed-fill), and an equivalent of ∼25 mm ha^-1^ water was applied at 10-days intervals. Soil analysis during the vegetative stage at both sites indicated that there were no nutrient deficiencies in soil in either year. Mature seeds at the R8 growth stage were collected, processed, and analyzed for seed composition constituent concentrations as described below.

### SOIL MINERALS, N, S, AND C ANALYSIS

Soil nutrient analyses were performed at the University of Georgia’s Soil, Plant, and Water Laboratory in Athens, GA. Concentrations of K were analyzed on a 5 g soil: 20 ml Mehlich-1 solution and the concentrations were determined using inductively coupled plasma spectrometry. Soil N, S, and C were determined by combusting samples using a C/N/S elemental analyzer having thermal conductivity cells (LECOCNS-2000 elemental analyzer, LECOCorporation, St. Joseph, MI, USA). A 0.25 g sample of soil was combusted in an oxygen atmosphere at 1350°C, converting elemental N, S, and C into N_2_, SO_2_, and CO_2_. The gasses were then passed through infrared cells and N, S, and C were determined by the elemental analyzer.

### LEAF AND SEED MINERALS, N, S, AND C ANALYSIS

Plant tissue samples were analyzed for different nutrients, including N, S, and C ratios. This was done by digesting 0.6 g of dried, ground plant materials in HNO_3_ in a microwave digestion system. Samples were ground using a Laboratory Mill 3600 (Perten, Springfield, IL, USA), and the concentration of K was determined using inductively coupled plasma spectrometry (Thermo Jarrell-Ash Model 61E ICP and Thermo Jarrell-Ash Autosampler 300; Bellaloui et al., 2011, 2014). For N, C, and S measurements, a 0.25 g ground-dried sample was combusted, and the percentages of C, N and S were determined using the C/N/S elemental analyzer (Bellaloui et al., 2011, 2014). For B, P, and Fe, concentrations were determined as described below.

### SEED ANALYSIS FOR PROTEIN, OIL, FATTY ACIDS, AND SUGARS

Mature seeds were analyzed for protein, oil, fatty acids, and sugars according to detailed methods as reported by Bellaloui et al. (2009b, 2010, 2014). Briefly, a 25 g sample of ground seed was analyzed for protein, oil, fatty acids, and sugars by near infrared reflectance (Wilcox and Shibles, 2001; Bellaloui et al., 2009b, 2010) using a diode array feed analyzer AD 7200 (Perten, Springfield, IL, USA). The calibration equation was initially developed by the University of Minnesota using Perten’s Thermo Galactic Grams PLS IQ software using conventional chemical protocols with AOAC methods (AOAC, 1990a,b). Then, the calibration equation for protein, oil, fatty acids, and sugars has been updated from 6 months to 1 year to insure accuracy and validity of the equation. Seeds at maturity were harvested at 13% water moisture and seed concentrations for protein, oil, fatty acids, and sugars were immediately performed on 13% water moisture and expressed on dry weight basis (Wilcox and Shibles, 2001; Boydak et al., 2002; Bellaloui et al., 2010, 2014). The fatty acid concentrations (palmitic, stearic, oleic, linoleic, and linolenic acids) were determined relative to total oils (Bellaloui et al., 2009b, 2014).

### SEED GLUCOSE AND FRUCTOSE ANALYSIS

The glucose level in seeds was measured by an enzymatic reaction using a Glucose (HK) Assay Kit, Product Code GAHK-20 (Sigma-Aldrich Co, St. Louis, MO, USA). A detailed description of the protocol and analysis was previously described by Bellaloui et al. (2014). Briefly, glucose and fructose were phosphorylated by adenosine triphosphate (ATP) and catalyzed by hexokinase, resulting in glucose-6-phosphate (G6P). The produced product (G6P) was then oxidized to 6-phosphogluconate by oxidized nicotinamide adenine dinucleotide (NAD) using glucose-6-phosphate dehydrogenase (G6PDH). The NAD was then reduced to NADH, and the concentration of glucose was determined based on the increase in absorbance at 340 nm. The seed samples involved were ground using the Laboratory Mill 3600, and a random sample of 0.1 mg was extracted with deionized water. The sample solution was heated to aid extraction, and a sample of 100 μl was added to 100 ml of the Glucose (HK) Assay Reagent and then incubated at room temperature for 15 min. A sample blank consisting of 100 and 1000 μl deionized water, and a reagent blank with 1000 μl of Glucose (HK) Assay Reagent and 100 μl of deionized water were used. Samples were read at an absorbance of 340 nm using the Beckman Coulter DU 800 spectrophotometer in order to determine glucose levels, which were expressed as mg g^-1^ dry weight.

Levels of fructose were measured using the method reported by Bellaloui et al. (2014). Fructose was phosphorylated by ATP using hexokinase, producing fructose 6-phosphate (F6P). The F6P produced was then converted to G6P by phosphoglucose isomerase (PGI), and the P6G then oxidized to 6-phosphogluconate in the presence of NAD in a reaction catalyzed by G6PDH. An equimolar amount of NAD was then reduced to NADH, and the concentration of fructose was measured by the same method as described for glucose.

### BORON DETERMINATION

Concentrations of boron in plant materials were measured using the azomethine-H method described by Lohse (1982) and Dordas et al. (2007), with a detailed description of the protocol reported by Bellaloui et al. (2014). Briefly, a ground sample of 1.0 g was ashed at 500^°^C, extracted with 20 ml of 2 M HCl at 90^°^C for 10 min, and then a 2 ml sample of the filtered mixture was added to 4 ml of buffer solution (containing 25% ammonium acetate, 1.5% EDTA, and 12.5% acetic acid). A volume of 4 ml of fresh azomethine-H solution (0.45% azomethine-H and 1% of ascorbic acid; John et al., 1975) was added. The concentrations of boron in leaves and seeds were determined at 420 nm using a Beckman Coulter DU 800 spectrophotometer (Beckman Coulter Inc., Brea, CA, USA).

### IRON DETERMINATION

Iron concentrations in leaves and seeds were determined according to Bandemer and Schaible (1944) and Loeppert and Inskeep (1996). The determination of the concentration was conducted by acid wet digestion, extraction, and reaction of the reduced ferrous Fe with 1,10-phenanthroline, as described by Bellaloui et al. (2011, 2014). Briefly, samples of 2 g of dried ground leaves and seeds were acid digested, and the soluble constituents were dissolved in 2 M of HCl. A volume of 4 ml of an aliquot containing 1–20 μg of iron of the sample solution was transferred into a 25 ml volumetric flask and diluted to 5 ml using 0.4 M HCl. A volume of 1 ml of Quinol solution was added to the 5 ml diluted sample solution and mixed. Three ml of the phenanthroline solution and 5 ml of the tri-sodium citrate solution (8% w/v) were added. The solution was diluted to 25 ml with distilled water and incubated at room temperature for 4 h. Phenanthroline reagent solution of 0.25% (w/v) in 25% (v/v) ethanol and quinol solution (1% w/v) was prepared. A standard curve was prepared using a concentration range from 0.0 to 4 μg ml^-1^ of Fe in 0.4 M HCl, with concentrations measured at an absorbance of 510 nm using the Beckman Coulter DU 800 spectrophotometer.

### PHOSPHORUS DETERMINATION

The yellow phosphor-vanado-molybdate complex method according to Cavell (1955) was used to determine phosphorus concentrations in leaves and seeds. The detailed description of the method was previously reported by Bellaloui et al. (2009b, 2014). Briefly, dried ground samples of 2 g of leaves and seed were ashed at 500°C, and 10 ml of 6 M HCl were added. The samples were placed in a water bath at 100°C to evaporate the solution to dryness. After the extraction of P using 2 ml of 36% v/v HCl under heat and filtration, 5 ml of 5M HCl and 5 ml of ammonium molybdate–ammonium metavanadate reagent were added to 5 ml of the filtrate. Ammonium molybdate–ammonium metavanadate was prepared by dissolving 25 g of ammonium molybdate and 1.25 g of ammonium metavanadate in 500 ml of distilled water. The phosphorus standard curve was established by preparing standard solutions of phosphorus in a range of concentrations from 0 to 50 μg ml^-1^ using dihydrogen orthophosphates. The concentrations of phosphorus were measured at an absorbance of 400 nm using the Beckman Coulter DU 800 spectrophotometer.

### EXPERIMENTAL DESIGN AND STATISTICAL ANALYSIS

The experimental design was a split-plot in a randomized complete block, with four replicates. A main plot was created for each PD, and subplots were a combination of either a single-row or twin-row planting with a SR of 20, 30, 40, or 50 seeds m^-2^. Replicates within years [rep (year)] and planting date × rep (year) were considered as components of variance for random effects. Year, PD, and SR were modeled as fixed effects. Residuals of random effect factors as covariance parameters were shown in tables; the residual values refer to Restricted Maximum Residual Likelihood (REML), which reflects the total variance of the random parameters in the model. Analysis of variance of data was performed using PROC MIXED in SAS (Statistical Analysis System, Copyright 2002–2010, Cary, NC, USA). Means were separated by Fisher’s protected LSD (0.05).

## RESULTS

### ANALYSIS OF VARIANCE (ANOVA) OF PLANTING DATE, ROW-TYPE, AND SEEDING RATE IN CLAY SOIL

In clay soil, ANOVA (**Tables 1** and **2**) showed that year (Y) and PD, and their interactions (Y × PD) had significant (ranged from *P* ≤ 0.05 to *P* ≤ 0.0001) effects on protein, oil, and fatty acids. RT had significant effects on protein, oil, stearic, and oleic acid, but not on palmitic, linoleic, and linolenic acids. However, interactions of PD × RT and Y × RT were significant for oil and oleic acid only, indicating that the response of oil and oleic acid to RT and PD were dependent on environmental factors in each year. Palmitic and stearic acids were the least affected by the interactions between Y, PD, and RT. Seeding rate had significant effects on protein, oil, and linoleic acid, while the interactions between SR, PD, and RT were significant for protein, oleic and linolenic acid. This indicated that the influence of SR on seed constituents was dependent on PD and RT. Based on this, the most sensitive constituents to agronomic practices and seasonal environmental factors were protein, oil, and oleic acid, and the least sensitive constituents to agronomic practices and environment were palmitic and stearic acids. Linoleic and linolenic acids were in between. The level of interactions between Y, PD, RT, and SR for seed composition ranged from *P* ≤ 0.05 to *P* ≤ 0.0001, depending on the seed constituent, indicating the different sensitivity of seed composition constituents to environments and agricultural practices (**Tables 1** and **2**). Except for raffinose, sugars and mineral concentrations were significantly influenced by Y and PD. However, Y × PD interactions were significant for B, P, and all sugars, except for stachyose. RT was significant for raffinose, glucose, fructose, B and P, while SR was significant for all minerals, and the sugars sucrose, raffinose, and glucose. Interactions between Y, PD, RT, and SR influenced some constituents. However, the least influenced constituent to agricultural practices was stachyose, while the most influenced constituents were the minerals (B, P, and Fe). Since Y interacted with other factors for some seed constituents, results were presented by year (Bellaloui et al., 2009b, 2010, 2011).

**Table 1:** Analysis of variance (*F* and *P* values) of seed protein, oil, fatty acids (g kg^**–1**^) in soybean as influenced by year (Y), seeding rate (SR), planting date (PD), and row-type (RT, single- or twin-row) in Sharkey clay soil in 2009 and 2010 at Stoneville, MS, USA.

		Protein		Oil		Palmitic		Stearic		Oleic		Linoleic		Linolenic	
Effect	DF	* F*	*P*	*F*	*P*	*F*	*P*	*F*	*P*	*F*	*P*	*F*	*P*	*F*	*P*
Y	1	5.9	*	96	***	275	***	0.34	NS	1130	***	759	***	53	***
PD	2	194	***	511	***	10.5	***	7.04	**	72	***	7.89	***	65	***
Y × PD	2	23.0	***	119	***	17.3	***	4.7	*	87	***	42.3	***	27	***
RT	1	36.3	***	7.8	**	0.1	NS	4.56	*	10.2	***	0.05	NS	0.64	NS
Y × RT	1	23.3	***	6.4	*	0.57	NS	3.18	NS	16.1	***	0.13	NS	3.7	NS
PD × RT	2	1.3	NS	5.6	**	0.75	NS	0.26	NS	10.6	***	0.3	NS	1.6	NS
Y × PD × RT	2	13.6	***	7.6	***	0.65	NS	0.08	NS	3.1	*	0.61	NS	2.7	NS
SR	3	6.4	***	4.1	**	0.55	NS	0.87	NS	2.2	NS	2.05	*	1.3	NS
Y × SR	3	1.7	NS	0.69	NS	1.4	NS	0.48	NS	1.4	NS	0.07	NS	2.4	NS
PD × SR	6	3.1	**	1.9	NS	0.63	NS	0.96	NS	2.4	*	3.8	**	1.4	NS
Y × PD × SR	6	1.9	NS	3.5	NS	0.70	NS	0.75	NS	3.6	**	1.2	NS	2.2	*
R × SR	3	1.5	NS	0.88	NS	3.8	NS	1.8	NS	2.6	NS	1.0	NS	0.69	NS
Y × RT × SR	3	0.26	NS	0.69	NS	1.8	NS	0.98	NS	1.6	NS	0.9	NS	0.61	NS
PD × RT × SR	6	2.3	*	0.82	NS	0.85	NS	0.65	NS	3.6	**	1.8	NS	3.1	**
Y × PD × RT × SR	6	2.2	*	1.0	NS	1.2	NS	1.0	NS	1.3	NS	1.3	NS	1.9	NS
Residual		35.8		21		41.3		1.4		109		90		32.1	

*P ≤ 0.05; **P ≤ 0.01; ***P ≤ 0.001; NS, not significant.

**Table 2:** Analysis of variance (*F* and *P* values) of sucrose, raffinose, stachyose, glucose, fructose (mg g^**–1**^), boron (B, mg kg^**–1**^), phosphorus (P, g kg^**–1**^), and iron (Fe, mg kg^**–1**^) in soybean as influenced by year (Y), seeding rate (SR), planting date (PD), and row-type (RT, single- or twin-row) in Sharkey clay soil in 2009 and 2010 at Stoneville, MS, USA.

		Suc		Raff		Stac		Glu		Fru		B		P		Fe	
Effect	DF	*F*	*P*	*F*	*P*	*F*	*P*	*F*	*P*	*F*	*P*	*F*	*P*	*F*	*P*	*F*	*P*
Y	1	187	***	210	***	837	***	271	***	12.0	***	11.4	**	1685	***	428	***
PD	2	33.6	***	2.4	NS	8.4	**	0.23	***	11.8	***	32.3	***	299	***	116	***
Y × PD	2	7.7	***	3.5	*	2.4	NS	0.32	***	15.1	***	18.7	***	17.4	***	0.76	NS
RT	1	75.1	NS	3.9	*	0.01	NS	102	***	400.8	***	8.5	**	21.0	***	3.0	NS
Y × RT	1	43.5	***	0.4	NS	1.2	NS	133	***	24.1	***	13.6	***	6.0	*	0.31	NS
PD × RT	2	1.4	NS	1.4	NS	1.6	NS	0.79	NS	37.1	***	0.31	NS	17.0	***	7.0	***
Y × PD × RT	2	1.4	NS	1.6	NS	1.2	NS	0.46	NS	54.8	***	0.99	NS	32.8	***	0.26	NS
SR	3	20.9	***	3.1	*	0.94	NS	27.4	***	0.68	NS	12.7	***	55.9	***	14.6	***
Year × SR	3	7.6	***	6.9	***	1.1	NS	27.3	***	1.5	NS	4.1	**	37.6	***	7.8	***
PD × SR	6	2.6	*	1.5	NS	0.52	NS	0.47	NS	2.8	**	1.4	NS	28.9	***	5.9	***
Y × PD × SR	6	1.9	NS	0.5	NS	0.96	NS	0.21	NS	3.2	**	1.9	NS	13.0	***	2.0	NS
R × SR	3	2.5	NS	8.4	***	0.83	NS	21.4	***	1.0	NS	10.3	***	25.5	***	9.2	***
Y × RT × SR	3	5.5	***	10.8	***	0.98	NS	25.5	***	2.1	NS	10.7	***	38.1	***	23.8	***
PD × RT × SR	6	1.7	NS	1.9	NS	2.6	NS	0.8	NS	0.9	NS	0.96	NS	7.0	***	0.87	NS
Y × PD × RT × SR	6	1.7	NS	2.4	*	1.7	NS	1.06	NS	3.0	**	2.4	*	4.6	***	2.3	*
Residual		16.3		0.08		10.6		0.16		0.012		21.8		0.04		12.4	

*P ≤ 0.05; **P ≤ 0.01; ***P ≤ 0.001; NS, not significant, suc, sucrose; raff, raffinose; stac, stachyose; glu, glucose; and fruc, fructose.

### ANALYSIS OF VARIANCE OF PLANTING DATE, ROW-TYPE, AND SEEDING RATE IN SANDY SOIL

In sandy soil, Y, PD, and their interactions (Y × PD) were significant (ranged from *P* ≤ 0.05 to *P* ≤ 0.0001) for protein, oil, and fatty acids, indicating that both Y and PD had different effects on these constituents, depending on the environmental factors in each year (**Table 3**). RT significantly interacted with Y and PD for the seed protein, oil, and palmitic acid, indicating that RT effects were dependent on Y and PD. However, RT on its own had no significant effects on these constituents. SR had significant effects on oil, oleic and linolenic acids, but its interaction (Y × SR) had significant effects on protein, oil, oleic, and linolenic acid. It can be concluded that under sandy soil conditions the most responsive constituents to agronomic practices and environment were protein, oil, and oleic acid, while the least responsive constituents were stearic, linoleic, and linolenic acids. Sugars, except glucose, were significantly influenced by both Y and PD (**Table 4**); however, interaction with Y did not affect sucrose, raffinose, or glucose. This showed that these sugars had the same response pattern in each year. Year, PD, and their interactions had significant effects on all minerals, indicating that these mineral levels were influenced by PD, but this influence is also affected by yearly environmental factors. RT had significant effects on all sugars (except glucose) and minerals, and its interaction with Y or PD was mainly significant for minerals. Sucrose, fructose, B and P were the constituents most influenced by SR and its interactions with Y and PD, reflecting the different response of seed constituents to agricultural practices such as PD, SR, and RT. It can be concluded that the least responsive constituents were stachyose, and glucose, and the most responsive were sucrose, fructose, and minerals (B, P, and Fe). Since Y interacted with other factors for some seed constituents, results were presented by year (Bellaloui et al., 2009b, 2010, 2011).

**Table 3:** Analysis of variance (*F* and *P* values) of seed protein, oil, fatty acids (g kg^**–1**^) in soybean as influenced by year (Y), seeding rate (SR), planting date (PD), and row-type (RT, single- or twin- row) in Beulah fine sandy loam soil in 2009 and 2010 at Stoneville, MS, USA.

Effect	DF	Protein		Oil		Palmitic		Stearic		Oleic		Linoleic		Linolenic	
Y	1	240	***	328	***	14.4	**	33.1	**	300	***	454	***	226	***
PD	2	105	***	123	***	243	***	37.9	***	4.95	*	64.1	***	0.59	NS
Y × PD	2	82.1	***	68.3	***	54.1	***	21.4	***	23	***	40.9	***	3.3	*
RT	1	0.01	NS	0.07	NS	0.26	NS	0.05	NS	2.3	NS	2.46	NS	0.04	NS
Y × RT	1	10.1	**	0.14	NS	17.3	***	0.18	NS	1.4	NS	0.24	NS	0.14	NS
PD × RT	2	11.6	***	9.4	**	8.7	**	0.58	NS	0.74	NS	0.81	NS	0.63	NS
Y × PD × RT	2	1.9	NS	10.3	***	15.4	***	0.52	NS	1.3	NS	0.7	NS	0.57	NS
SR	3	0.24	NS	14.8	***	1.3	NS	0.51	NS	28.9	***	0.15	NS	27.7	***
Year × SR	3	3.7	**	6.3	**	0.79	NS	1.17	NS	30.1	***	0.18	NS	31.9	***
PD × SR	6	2.4	*	12.2	***	3.2	**	0.81	NS	2.1	NS	0.52	NS	1.3	NS
Y × PD × SR	6	2.5	*	17.4	***	4.4	**	1.01	NS	1.6	NS	0.48	NS	1.6	NS
RT × SR	3	2.3	NS	7.1	**	1.0	NS	0.03	NS	0.67	NS	0.19	NS	1.1	NS
Y × RT × SR	3	1.36	NS	7.1	**	3.8	*	0.92	NS	0.61	NS	0.21	NS	1.0	NS
PD × RT × SR	6	3.9	NS	5.9	***	4.03	**	0.83	NS	3.47	**	1.51	NS	0.66	NS
Y × PD × RT × SR	6	5.53	***	3.4	**	1.94	NS	0.8	NS	3.23	**	0.43	NS	0.35	NS
Residual		53.6		28.1		29.6		1.92		155		161		38.1	***

*P ≤ 0.05; **P ≤ 0.01; ***P ≤ 0.001; NS, not significant.

**Table 4:** Analysis of variance (*F* and *P* values) of sucrose, raffinose, stachyose, glucose, fructose (mg g^**–1**^), and boron (B, mg kg^**–1**^), phosphorus (P, g kg^**–1**^), and iron (Fe, mg kg^**–1**^) in soybean as influenced by year (Y), seeding rate (SR), planting date (PD), and row-type (RT, single- or twin-row) in Beulah fine sandy loam soil in 2009 and 2010 at Stoneville, MS, USA.

		Suc		Raff		Stac		Glu		Fru		B		P		B	
Effect	DF	*F*	*P*	*F*	*P*	*F*	*P*	*F*	*P*	*F*	*P*	*F*	*P*	*F*	*P*	*F*	*P*
Y	1	500.	***	374	***	153	***	665	***	5216	***	442	***	1044	***	489	***
PD	2	13.8	**	14.3	**	12.8	**	0.02	NS	22.41	***	75.6	***	79.9	***	9.2	**
Y × PD	2	2.59	NS	2.3	NS	6.58	**	0.5	NS	2.71	NS	44.0	***	79.75	***	9.7	**
RT	1	132	***	23.6	***	12.5	**	669	NS	407	***	150	***	162	***	132	***
Y × RT	1	2.9	NS	57.6	***	0.4	NS	2.26	NS	28.8	***	7.17	**	28.6	***	54.7	***
PD × RT	2	2.3	NS	1.47	NS	0.07	NS	0.56	NS	64.0	***	2.79	NS	19.3	***	17.8	***
Y × PD × RT	2	1.3	NS	0.1	NS	1.86	NS	1.46	NS	26.8	***	0.92	NS	17.8	***	14.2	***
SR	3	8.6	***	0.72	NS	0.61	NS	1.4	NS	5.41	**	54.7	***	65.3	***	1.06	NS
Year × SR	3	2.6	*	0.89	NS	0.86	NS	0.0	NS	0.0	NS	0.0	NS	0.0	NS	0.29	NS
PD × SR	6	3.5	**	5.5	***	0.59	NS	0.38	NS	5.1	***	22.91	***	31.0	***	2.8	**
Y × PD × SR	6	1.5	NS	2.29	*	0.45	NS	0.41	NS	6.9	***	18.75	***	61.4	***	4.93	***
R × SR	3	3.6	NS	0.44	NS	0.88	NS	0.87	NS	5.1	**	12.48	***	88.5	***	6.8	**
Y × RT × SR	3	0.7	NS	0.54	NS	1.27	NS	0.0	NS	0.0	NS	0.0	NS	0.03	NS	2.5	NS
PD × RT × SR	6	3.87	**	4.53	**	1.9	NS	0.86	NS	11.6	***	0.93	NS	9.6	***	17.5	***
Y × PD × RT × SR	6	5.1	***	5.6	***	0.27	NS	0.62	NS	17.4	***	0.52	NS	12.2	***	4.9	**
Residual		20		0.25		13.9		0.14		0.01		9		0.06		12.8	

*P ≤ 0.05; **P ≤ 0.01; ***P ≤ 0.001; NS, not significant; suc, sucrose, raff, raffinose; stac, stachyose; glu, glucose; and fruc, fructose.

### EFFECTS OF PLANTING DATE, SEEDING RATE, AND ROW-TYPE ON SEED COMPOSITION IN CLAY SOIL

Mean values in 2009 in clay soil and in April planting (**Table 5**) showed that protein concentrations decreased with increasing SR on single-rows, but increased on twin-rows. On the single-rows, the linolenic acid was reduced with SR, but remained constant or not consistent on the twin-rows. Oleic acid, sucrose, P, and B increased with increasing SR, but this increase continued only until a maximum concentration reached, after which the concentration deceased or remained constant. No consistent effects of SR increase were observed for the other seed constituents. A similar pattern was observed for B and P in May and June plantings on single- and twin-rows. However, for these PDs protein concentration increased with SR on single-row, but decreased on twin-row (**Table 5**). Linolenic acid increased with SR and then decreased at higher SR on single- and twin-rows. In May planting the concentrations of oil and minerals increased with SR increases on single- and twin-rows, but the pattern of oleic and protein varied, depending on RT. Generally, protein was higher in May and June plantings than in April planting, but oil concentration had the opposite trend, higher in April and lower in May and June plantings on single- or twin-rows. In 2010, except in May planting, protein, glucose, B, and Fe concentrations increased with SR increase in April and June plantings (**Table 6**) on single- and twin-rows. Protein was higher in May planting and oil was higher in April planting on single- and twin-rows, confirming the observation in 2009. Oleic acid was higher in 2010 than in 2009 for all PDs and on single- and twin-rows.

**Table 5:** Effects of row-type (RT, single, S or twin, T), seeding rate (SR, seed m^**–**2^), and planting date on seed protein, oil, fatty acids (g kg^**–1**^), sucrose (Suc), stachyose (Stac), glucose (Glu) (mg g^**–1**^), boron (B, mg kg^**–1**^), phosphorus (P, g kg^**–1**^), and iron (Fe, mg kg^**–1**^) in soybean in Sharkey clay soil in 2009 at Stoneville, MS, USA.

Planting	RT	SR	Protein	Oil	Oleic	Linolenic	Suc	Stac	Glu	B	P	Fe
April		20	420	242	231	67.6	46.5	26.7	2.8	38.3	4.5	66.0
	S	30	413	242	237	66.4	47.2	26.3	2.8	44.7	4.5	66.0
		40	419	237	246	56.7	47.2	29.4	2.6	43.8	5.6	65.5
		50	417	242	256	56.3	45.9	26.3	2.8	36.4	4.5	63.8
		LSD	1.90	2.10	4.20	2.90	1.21	0.70	0.20	3.70	0.07	1.30
		20	409	245	246	60.0	52.8	28.3	4.1	41.1	4.6	57.0
	T	30	408	243	281	61.3	61.5	27.2	4.1	47.2	5.6	66.8
		40	408	244	275	61.5	62.5	26.8	4.1	48.2	5.6	73.3
		50	410	241	266	61.5	46.0	28.0	4.0	48.5	4.3	61.0
		LSD	3.50	2.70	5.00	2.30	1.10	1.10	0.21	1.30	0.08	1.40
May		20	449	217	222	75.4	48.7	23.5	2.9	29.1	3.5	64.0
	S	30	454	215	223	80.3	47.2	22.1	3.1	30.0	3.4	62.5
		40	460	209	218	74.2	48.1	23.7	2.8	34.6	4.4	63.8
		50	458	203	220	77.8	45.5	25.1	2.6	32.0	4.6	60.0
		LSD	2.30	2.00	3.30	3.50	0.81	1.00	0.16	1.90	0.09	1.90
		20	458	203	224	75.9	53.8	24.2	4.1	28.9	4.4	53.0
	T	30	452	206	245	88.5	61.0	23.7	4.0	29.5	5.3	64.5
		40	447	201	230	73.9	62.3	21.8	4.3	38.9	4.6	64.0
		50	452	201	211	71.8	52.0	25.4	4.2	38.4	4.3	54.0
		LSD	4.40	2.90	4.70	2.90	0.91	1.20	0.19	4.50	0.18	1.20
June		20	439	223	204	72.7	55.8	24.2	2.7	26.6	3.4	52.5
	S	30	449	223	207	76.3	51.3	24.7	2.9	34.1	3.5	55.5
		40	441	223	207	81.6	55.3	23.4	3.2	31.4	4.3	53.8
		50	446	227	211	78.8	45.5	26.5	2.7	30.5	4.6	58.8
		LSD	2.40	2.90	3.90	3.60	1.00	1.10	0.16	1.30	0.15	1.70
		20	419	217	220	90.6	59.0	24.5	4.0	28.8	3.7	45.8
	T	30	434	220	201	79.9	63.0	26.4	4.1	37.8	3.5	57.8
		40	433	217	205	82.2	62.0	23.8	4.1	38.5	3.7	63.3
		50	422	224	209	83.7	54.8	27.3	4.1	39.2	3.7	57.8
		LSD	4.10	2.60	7.70	3.40	2.30	1.20	0.20	1.50	0.09	2.70

Means were separated by Fisher’s least significant difference LSD (0.05).

**Table 6:** Effects of row-type (RT, single, S or twin, T), seeding rate (SR, seed m^**–**2^), and planting date on seed protein, oil, fatty acids (g kg^**–**1^), sucrose (Suc), stachyose (Stac), glucose (Glu) (mg g^**–**1^), boron (B, mg kg^**–**1^), phosphorus (P, g kg^**–**1^), and iron (Fe, mg kg^**–**1^) in soybean in Sharkey clay soil in 2010 at Stoneville, MS, USA.

Planting	RT	SR	Protein	Oil	Oleic	Linolenic	Suc	Stac	Glu	B	P	Fe
April		20	419	231	279	66.3	23.8	38.3	1.6	28.1	3.6	47.8
	S	30	424	227	276	62.5	23.3	39.3	1.8	36.8	3.4	57.0
		40	421	230	282	60.7	23.8	42.3	2.6	33.9	3.5	52.8
		50	423	228	275	69.7	25.3	42.8	3.4	42.1	3.4	57.3
		LSD	2.80	1.90	4.70	2.40	3.40	2.20	0.21	1.30	0.10	1.40
		20	411	231	278	59.3	23.5	41.8	2.1	37.2	3.6	54.3
	T	30	423	226	289	64.2	21.5	44.3	2.0	27.0	3.4	53.0
		40	421	230	270	63.8	24.0	40.8	3.4	45.3	3.4	55.5
		50	421	228	284	66.1	22.0	40.5	2.3	30.5	3.3	55.0
		LSD	2.60	1.90	4.90	2.40	2.90	1.70	0.30	5.00	0.05	1.30
May		20	442	215	281	64.5	26.0	42.5	1.9	29.9	3.3	48.3
	S	30	437	215	275	65.8	26.0	39.8	2.0	37.2	2.2	57.0
		40	445	214	277	66.4	35.5	43.3	2.5	32.1	3.2	53.3
		50	443	212	289	70.7	27.5	40.3	3.8	34.3	3.4	52.5
		LSD	2.41	1.50	5.60	2.80	1.80	2.00	0.14	1.20	0.13	1.50
		20	437	217	278	68.5	33.3	37.5	1.9	36.8	3.4	56.0
	T	30	438	215	287	66.8	24.5	37.8	1.9	26.3	2.3	44.0
		40	437	216	284	68.2	34.3	38.3	3.7	35.3	3.3	54.0
		50	440	216	282	65.5	33.5	42.0	1.7	35.5	3.4	43.5
		LSD	2.20	1.70	7.60	2.40	1.60	1.90	0.19		0.07	1.50
June		20	419	210	286	72.4	35.3	42.3	1.8	29.6	2.4	41.5
	S	30	431	213	295	66.8	29.3	40.3	1.6	36.8	2.5	43.8
		40	436	203	276	68.7	26.3	38.0	2.7	35.2	2.4	43.5
		50	438	206	284	69.8	25.5	39.0	3.5	37.0	3.4	47.8
			4.70	2.20	4.90	2.20	2.10	1.80	0.20	1.10	0.08	2.30
		20	430	210	278	69.0	32.3	36.5	2.0	34.8	2.2	45.3
	T	30	437	207	275	69.4	32.0	38.8	1.9	24.8	2.5	43.8
		40	435	203	276	65.1	36.3	44.3	3.5	34.0	3.3	43.8
		50	437	203	278	67.5	24.8	38.8	1.7	39.3	3.5	47.0
		LSD	2.60	2.10	2.80	2.60	2.70	2.40	2.19	1.60	0.11	1.40

Means were separated by Fisher’s least significant difference LSD (0.05).

### EFFECTS OF PLANTING DATE, SEEDING RATE, AND ROW-TYPE ON SEED COMPOSITION IN SANDY SOIL

Mean values in 2009 (**Table 7**) showed that in April planting on single-rows, oleic acid decreased and linolenic acid and minerals increased with increasing SR until the maximum concentration was achieved, after which the concentrations either became constant or declined. On twin-rows, protein, linolenic acid, sucrose, B, and P concentrations increased with increasing SR, and oleic acid and stachyose decreased. In May planting, protein, oil, linolenic acid, B, and P increased with increasing SR on single-rows. On twin-rows, linolenic acid, sucrose, glucose, B, and P concentrations increased, and protein and oleic acid concentrations decreased. In June planting on single-rows, linolenic acid, glucose, B, P, and Fe concentrations increased with increasing SR, but oleic acid decreased. On twin-rows, oleic acid decreased and linolenic acid, sucrose, and B concentrations increased. Protein was higher in June planting, but oil was higher in April planting. In 2010 (**Table 8**) in April planting, sucrose and Fe concentrations increased with increasing SR on single- and twin-rows. In May planting, protein concentration decreased with SR, but oil concentration and B concentrations increased. In June planting, protein, P, and Fe increased and oil and oleic acid decreased with SR on single- and twin-rows. Sucrose and B concentrations showed a pattern of increase with increasing SR on twin-rows.

**Table 7:** Effects of row-type (RT, single, S or twin, T), seeding rate (SR, seed m^**–**2^), and planting date on seed protein, oil, fatty acids (g kg^**–**1^), sucrose (Suc), stachyose (Stac), glucose (Glu) (mg g^**–**1^), boron (B, mg kg^**–**1^), phosphorus (P, g kg^**–**1^), and iron (Fe, mg kg^**–**1^) in soybean in Beulah fine sandy loam soil in 2009 at Stoneville, MS, USA.

Planting	RT	SR	Protein	Oil	Oleic	Linolenic	Suc	Stac	Glu	B	P	Fe
April		20	419	231	258	63.1	55.0	23.1	4.1	42.7	5.0	60.6
	S	30	419	223	270	66.6	54.5	26.4	4.1	48.2	5.0	65.5
		40	421	234	246	73.6	54.8	25.8	3.9	48.1	6.0	71.5
		50	419	236	220	81.3	55.3	27.4	4.1	40.8	4.9	55.0
		LSD	2.60	2.40	2.90	3.90	0.93	1.30	0.20	1.20	0.07	1.70
		20	416	229	264	68.2	60.3	28.0	5.6	46.7	5.4	65.5
	T	30	424	230	254	65.5	65.3	26.4	5.7	52.8	6.5	58.5
		40	422	230	239	84.7	66.3	27.7	5.6	53.8	6.5	63.3
		50	422	233	252	79.1	67.5	25.1	5.5	54.1	5.2	67.0
		LSD	5.00	3.60	6.50	5.20	3.00	1.30	0.21	1.30	0.08	1.90
May		20	448	208	269	67.4	55.3	19.0	4.2	33.4	3.9	62.8
	S	30	457	208	275	63.8	60.5	17.8	4.4	34.3	3.8	56.3
		40	460	211	231	76.3	60.8	18.8	4.1	38.9	4.9	64.0
		50	466	212	224	80.3	57.3	16.4	3.9	36.4	5.0	65.8
		LSD	5.10	2.60	4.50	3.30	2.60	2.30	0.16	1.90	0.08	1.90
		20	469	215	279	63.2	58.0	18.9	5.6	34.5	5.3	63.8
	T	30	450	216	270	58.8	72.0	20.2	5.5	42.9	6.1	57.5
		40	437	206	254	83.1	75.8	22.9	5.8	44.5	5.5	56.5
		50	456	212	242	80.8	63.3	22.2	5.7	44.0	5.2	55.0
		LSD	5.90	2.00	5.30	2.00	2.60	2.00	0.19	1.40	0.18	2.00
June		20	463	213	291	57.9	61.3	16.4	4.0	30.9	3.9	55.8
	S	30	460	213	281	63.8	63.8	18.9	4.0	38.4	4.0	65.0
		40	451	211	236	80.6	63.8	18.2	4.2	35.7	4.7	55.5
		50	466	211	263	81.6	62.8	18.3	4.5	34.8	5.1	65.5
		LSD	3.50	2.30	6.90	3.20	1.80	1.80	0.16	1.30	0.15	0.81
		20	454	213	287	58.5	64.8	20.8	5.6	34.4	4.5	74.0
	T	30	447	207	297	62.1	70.8	21.5	5.6	43.4	4.4	72.0
		40	462	209	245	79.5	72.5	18.4	5.6	44.1	4.5	71.0
		50	452	211	240	79.1	71.0	21.4	5.6	44.8	4.5	64.5
		LSD	3.00	2.90	3.60	1.90	1.90	1.70	0.20	1.50	0.09	2.20

*Means were separated by Fisher’s least significant difference LSD (0.05). Suc, sucrose; stac, stachyose, and glu, glucose*.

**Table 8:** Effects of row-type (RT, single, S or twin, T), seeding rate (SR, seed m^**–**2^), and planting date on seed protein, oil, fatty acids (g kg^**–**1^), sucrose (Suc), stachyose (Stac), glucose (Glu) (mg g^**–**1^), boron (B, mg kg^**–**1^), phosphorus (P, g kg^**–**1^), and iron (Fe, mg kg^**–**1^) in soybean in Beulah fine sandy loam soil in 2010 at Stoneville, MS, USA.

Planting	RT	SR	Protein	Oil	Oleic	Linolenic	Suc	Stac	Glu	B	P	Fe
April		20	408	226	280	57.3	41.5	30.0	2.9	34.7	4.0	44.5
	S	30	419	194	289	58.4	42.8	28.3	2.8	26.4	2.9	50.5
		40	423	197	296	59.5	49.3	30.3	2.5	27.8	3.7	49.3
		50	415	226	300	55.6	40.3	32.3	2.5	32.8	4.0	44.5
		LSD	3.30	2.90	6.70	2.90	2.18	2.50	0.18	1.50	0.16	1.51
		20	430	205	301	58.4	46.8	33.5	3.8	36.5	4.1	56.0
	T	30	423	204	289	57.5	49.8	35.3	3.8	32.6	4.8	54.8
		40	433	197	287	58.1	48.3	34.3	3.8	33.9	3.2	55.5
		50	431	199	272	56.5	49.8	30.8	3.7	43.9	3.8	64.8
		LSD	2.90	2.50	7.4	2.10	2.40	1.96	0.20	1.69	0.10	0.67
May		20	423	191	306	57.6	37.5	33.0	2.6	25.5	2.9	50.8
	S	30	425	202	302	55.7	50.8	26.8	2.6	30.5	3.0	46.8
		40	420	206	302	57.3	40.3	30.0	2.3	40.2	5.1	44.3
		50	413	230	296	56.8	37.5	29.5	2.3	28.4	4.1	44.8
		LSD	2.70	2.00	7.10	2.90	2.00	2.20	0.18	1.00	0.08	1.90
		20	421	202	296	58.1	50.3	30.0	3.8	24.3	4.0	52.8
	T	30	418	201	302	51.7	49.5	28.5	3.8	33.1	3.0	55.5
		40	420	211	300	59.8	54.0	32.0	3.8	43.6	5.2	57.3
		50	408	220	306	57.2	49.5	30.0	4.0	33.7	3.8	56.3
		LSD	3.60	3.30	3.60	2.40	2.30	1.80	0.18	1.00	0.18	1.60
June		20	429	197	287	63.1	52.5	29.0	2.4	23.0	2.9	42.3
	S	30	431	196	286	60.6	40.0	29.8	2.5	40.2	4.0	45.0
		40	433	190	284	56.4	42.3	32.5	2.5	31.0	3.9	48.8
		50	430	191	279	61.5	51.0	31.5	2.4	26.9	4.1	47.0
		LSD	1.84	1.60	6.20	2.80	2.10	2.20	0.16	1.90	0.07	2.30
		20	426	200	288	62.3	46.3	32.8	3.8	24.1	3.2	63.5
	T	30	429	206	283	61.7	57.5	32.8	3.9	42.5	5.1	54.8
		40	433	198	289	54.4	49.8	33.0	4.1	34.2	4.2	54.8
		50	437	195	279	62.1	50.0	28.8	3.8	34.6	3.2	49.8
		LSD	1.60	2.90	7.60	2.80	2.40	2.20	0.22	1.50	0.07	2.00

*Means were separated by Fisher’s least significant difference LSD (0.05). Suc, sucrose; stac, stachyose; and glu, glucose*.

### SOIL AND LEAF NUTRIENTS LEVELS

Soil analyses showed that in clay soil, the average nutrient levels in 2009 and 2010, respectively, were: C = 1.40 and 1.10%; N = 0.13 and 0.10%; S = 31.2 and 30.9 mg kg^-1^; K = 2340 and 2570 mg kg^-1^; P = 355 and 368 mg kg^-1^; B = 2.45 and 2.92 mg kg^-1^; and Fe = 20.13 and 22.91 g kg^-1^. In sandy soil, the average nutrient levels, respectively, in 2009 and 2010 were: C = 1.10 and 1.30%; N = 0.10 and 0.11%; S = 28.7 and 30.1 mg kg^-1^; K = 2140 and 2060 mg kg^-1^; P = 267 and 284 mg kg^-1^; B = 1.20 and 1.60 mg kg^-1^; and Fe = 18.72 and 18.89 g kg^-1^. Nutrient concentrations in leaf samples in clay soil, respectively in 2009 and 2010 were: N = 5.20 and 4.11%; S = 0.31 and 0.27%; K = 2000 and 1570 mg kg^-1^; P = 390 and 230 mg kg^-1^; B = 40.56 and 33.76 mg kg^-1^; and Fe = 209 and 86 mg kg^-1^. Nutrient concentrations in leaves in sandy loam soil, respectively in 2009 and 2010, were: N = 4.80 and 4.76%; S = 0.37 and 0.23%; K = 2.20 and 1.43%; P = 0.41 and 0.29%; B = 35.7 and 28.70 mg kg^-1^; and Fe = 157 and 94.50 mg kg^-1^. The analysis of random samples of fully expanded leaves taken across the field at R5 to R6 showed adequate concentrations of nutrients in soybean grown in both sites. It was noticed that concentrations of nutrients, especially for K, B, P, and Fe, in 2009 were greater than in 2010 and this observation was attributed to heat and drier conditions in 2010.

## DISCUSSION

### EFFECTS OF PLANTING DATE, SEEDING RATE, AND ROW-TYPE ON SEED COMPOSITION

The current research showed that April planting (early planting) resulted in higher oil, oleic acid, sucrose, and minerals, especially B and P, on both single and twin-rows, and June planting (late planting) resulted in higher protein and linolenic acid, but lower oleic acid and oil concentrations in 2009 and 2010. Previous research showed that the response of seed constituents to PD was mainly due to temperature differences (Dardanelli et al., 2006; Bellaloui et al., 2009b) or other environmental factors such as drought (Piper and Boote, 1999; Bellaloui et al., 2009b, 2014). Dardanelli et al. (2006) investigated the consistency of soybean MG effects and their interactions with the environment on protein and oil, and found that environment was mainly defined by PD and location, and that environment was the most important source of variation for protein and oil. Previous research on the effects of PD on seed composition is still inconsistent and inconclusive. For example, oil concentration increased with early planting, but this increase pattern was not consistent across locations (Helms et al., 1990; Kane et al., 1997; Pedersen and Lauer, 2004), indicating that the response of seed constituents to PDs depends on the environment under which soybeans are grown.

Our research showed that late planting (June planting) resulted in the increase of protein concentration and decrease in oil concentration, partially agreeing with those of Helms et al. (1990) and Pedersen and Lauer (2004). The high oleic acid and low linolenic acid concentrations in the early planting (April planting), and the low oleic acid and high linolenic acid in late planting, especially the June planting, could be due to the inverse relationship between these two constituents (Carver et al., 1986; Dornbos and Mullen, 1992; Bellaloui et al., 2009b) and temperature differences. Studying high oleic acid gemplasm lines, it was suggested that the instability of oleic acid across environments was due mainly to temperature effects on enzymes controlling biosynthesis of soybean seed fatty acids, especially during seed-fill (R5 to R6) stage (Howell and Collins, 1957; Wilcox and Cavins, 1992; Bachlava and Cardinal, 2009). Bachlava and Cardinal (2009) suggested that the late planting may result in a decrease of linolenic and palmitic acids, but stearic acid may increase, and the changes in fatty acids with late planting may be due to temperature changes during seed maturation at later planting (Wilcox and Cavins, 1992). Other researchers showed that high oleic and low linolenic acids under warmer conditions (Carver et al., 1986) were explained as consequences of the effects of temperature on oleic acid and linoleic acid desaturases (Burton, 1991). These effects include a decrease in oleic- and linoleic-desaturase activities at 35°C (Cheesbrough, 1989), a decrease in ω-6 desaturase enzyme (encoded by the *FAD2-1A* gene), and the degradation of desaturases at high growth temperatures of 30°C (Tang et al., 2005). Another possible explanation for high linolenic and low oleic acids in late planting is that late planting coincides with a cooler temperature that favors linolenic acid accumulation and lowering of oleic acid. This explanation could be supported by our findings that linolenic acid concentration increased with increased SR for all PDs in sandy soil in 2009. This may be due to shade effects and cooler temperatures in the lower plant canopy which resulted from higher plant densities at higher SR (40 and 50 seed m^-2^). Previous research investigated the effect of upper and lower plant parts on seed nutrients, including seed oil and protein (Collins and Cartter, 1956), protein (Escalante and Wilcox, 1993), protein, oil, oleic, and linolenic acids (Bellaloui and Gillen, 2010; Bellaloui et al., 2012). They found that seed located at the top central portion had lower seed oil and higher protein contents (Collins and Cartter, 1956), while Kochegura (1982) found that seeds from the higher plant canopy had higher seed protein than seeds at lower plant canopy. Other researchers found that there were no differences between the upper and lower canopy (Huskey et al., 1990). Bellaloui et al. (2012) found that the lower plant canopy had higher oil and linolenic acid, but lower protein and oleic acid. The differences in seed oil between the upper and lower plant canopy was explained to be due to environmental differences during seed oil synthesis and accumulation (Collins and Cartter, 1956). Others reported that the higher seed oil and linolenic acid and the lower protein and oleic acids in seeds in the upper plant canopy was due to cooler temperature and shade effects as cooler temperature enhances linolenic acid and oil (Bellaloui et al., 2012). Therefore, the higher linolenic acid concentration with increasing SR and late planting could be due to shade effects and cooler temperature. Since linolenic acid and oleic acid showed to have an inverse relationship (Bellaloui et al., 2009a,b), the lower oleic acid is a consequences of the higher linolenic acid.

**FIGURE 1:**
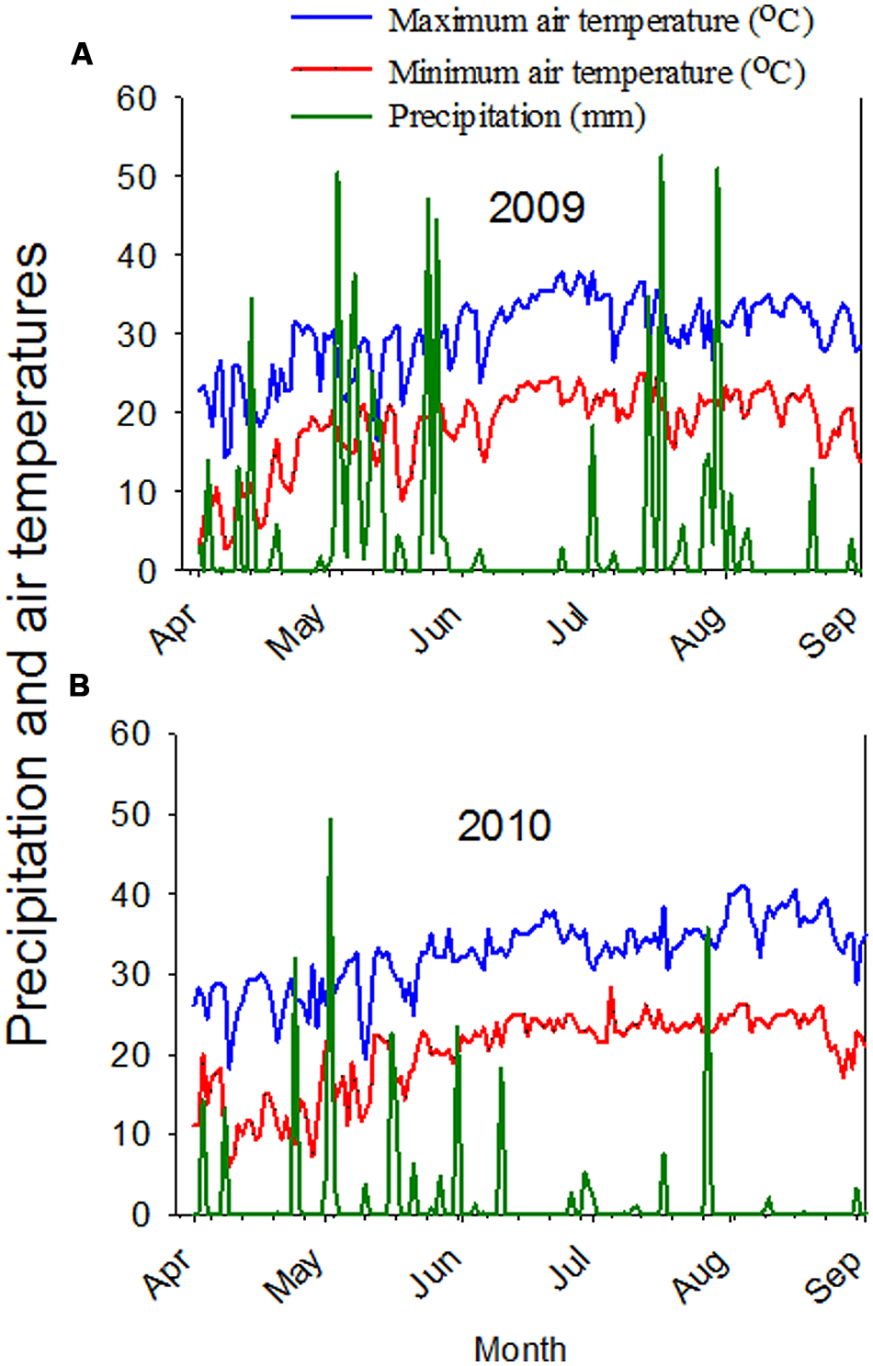
**Maximum and minimum air temperatures, and precipitation in 2009 **(A)** and in 2010 **(B)**.** Weather data obtained from MSUCares, Stoneville, MS, USA, 2014, (http://ext.msstate.edu/anr/drec/weather.cgi).

Increasing protein and B with increasing SR in 2009 in sandy and clay soils, especially for May and June plantings, increasing linolenic acid in sandy soil in April planting, and increasing sucrose in 2009 in both clay and sandy soils indicated the positive response of these constituents to SR increases under these conditions. The higher concentrations of B and sucrose in twin-rows, compared to single-rows, for all PDs in 2009 can be explained in terms of light interception and row-spacing effects. Twin-rows resulted in narrow-plantings, while single-row plantings were wider. It was reported that narrow-row soybean had higher canopy radiation interception than wider rows (Shibles and Weber, 1966; Taylor et al., 1982), and differences in radiation interception was observed between narrow-row and wide-rows during the period from R6 (beginning seed-fill) to R7 (full seed-fill) stages (Fehr and Caviness, 1977). These differences were attributed to leaf area distribution and duration (Taylor et al., 1982). Other researchers reported that the reasons for higher yield in narrower rows (from 25 to 50 cm) compared to wider rows (Bharati, 1977; Cooper, 1977) was usually attributed to the development of a full canopy (95% light interception) before rapid seed development (Shibles and Weber, 1966), resulting in greater photosynthetic rate (Shibles and Weber, 1965), and nutrient uptake and translocation (Bellaloui et al., 2014). Therefore, the higher concentrations of sucrose and B in twin-rows could be due to the possible association between sucrose and B nutrition and light interception and photosynthesis. The higher concentrations of oleic acid in 2010, especially in clay soil, may be due to higher temperature and a drier year in 2010 (**Figure 1**). The higher concentrations of stachyose in 2010 may be due to a drier year and higher temperature in 2010 compared with 2009, suggesting a possible role of stachyose as an environmental stress compound under drought and high temperature. Although the biological functions of stachyose are still not clear (Ren et al., 2009), Obendorf (1997) reported that oligosaccharides, including stachyose, are required for the acquisition of desiccation tolerance during seed development and maturation, and could be involved in seed protection against damage during seed dehydration to ensure seed survival and storability.

Very limited information on the effects of SR and row-spacing on seed composition was reported, and what is available is on other species (Bellaloui et al., 2014). For example, effect of SR and row-spacing on seed protein and oil in rapeseed (*Brassica napus* L.) had no consistent effects (Kondra, 1975). Other researchers evaluated the effects of three row-spacing (30, 40, and 50 cm) on canola seed oil and found there was no relationship between row-spacing and oil, but the highest oil concentration was recorded at row-space of 30 cm (Mousavi et al., 2011). Studying the effects of SRs of 60, 50, 40, and 30 plants m^-2^ on saﬄower (*Carthamus tinctorius* L.) oil under irrigated and rainfed conditions, Amoghein et al. (2012) found that the highest oil percentage was achieved by the lowest SR of 30 plants m^-2^, but no oil differences was observed between 40, 50, and 60 plant m^-2^. SR from 30,000 to 45,000 plants ha^-1^ resulted in higher oil in Sunflower (*Helianthus annuus* L.), while SR beyond 45,000 plants ha^-1^ resulted in a smaller increases (Gubbels and Dedio, 1986). Recently, Bellaloui et al. (2014) investigated the effects of SR and row-spacing on seed protein, oil, fatty acids, sugars, and minerals using four soybean cultivars (P 93M90, AG 3906, P 94B73, and V 52N3) in 2006 and 2007 in the Midsouth USA. They found that protein, oleic acid, sugars, P, and B concentrations increased with the increase of SR in P 93M90 and AG 3906, but the concentrations of these constituents decreased after the maximum concentrations were reached, supporting our results. The pattern of increase was mainly observed in 2006 and depended on the row-spacing. In 2007, however, the opposite trend (protein and oleic decreased with SR) was noticed. In cultivars P 94B73 and V 52N3 protein concentration increased with SR in 2006 and 2007 for both 38 and 76 cm row-spacing. An increase of oleic acid and a decrease of linolenic acid with row-spacing were observed in 2006. They concluded that SRs and row-spacing can alter some seed composition constituents, and the effect of SRs depended on the rate used, row-spacing, genotype, and the growing conditions of each year. They also reported that the different pattern (positive or negative) of seed constituents between years due to the environmental factors such as temperature and drought.

The response of some seed constituents to PD, SR, or RT was different between 2009 and 2010, and this may be due to high heat and drier year in 2010. Weather data showed that the average maximum temperature reached 34°C in July and 37°C in August in 2010 compared with 32.2 and 34.4°C in July and August, respectively in 2009 (**Figure 1**; MSUCares, 2014). The precipitation in July was 46.0 and 6.1 mm in August in 2010, but was 203.7 mm in July and 36.1 mm in August in 2009 (**Figure 1**; MSUCares, 2014). Previous research showed that relationships between seed composition constituents differ between years due to heat and drought (Bellaloui et al., 2009b, 2014). Our soil analysis showed that both clay and sandy soils had adequate nutrient levels; however, leaf samples collected in 2009 and 2010 showed that leaf nutrient levels in 2010 were lower than in 2009, and this may be due to high heat and drier year in 2010, affecting the concentrations of seed composition and minerals.

We did not observe clear patterns for protein, oil, or fatty acids between single- and twin-rows, although other researchers found that row-spacing and irrigation significantly affected protein and oil contents, and that row-spacing (RS) of 70 cm had the highest protein content, followed by RS of 60, 40, and 50 cm, respectively (Boydak et al., 2002). They also found that RS had a significant (*P* < 0.01) influence on oleic and linoleic acid content, and a row-spacing of 50 cm produced maximum oil value, but a row-spacing of 70 cm produced the highest protein value (39.05%), and 50 cm produced the lowest value (37.65%).

## CONCLUSION

The current research showed that early planting resulted in higher soybean seed oil and oleic acid, but lower protein and linolenic acid concentrations. The late planting resulted in higher protein and linolenic acid. These changes in seed constituents were attributed mainly to temperature changes and drought, indicating that shifts in PDs create a new environment. Concentrations of protein, linolenic acid, sucrose and B increased with SR increases, possibly due to higher light interception and early canopy closure. The SRs that resulted in highest levels of seed constituents (for example, for protein, oil, oleic and linolenic acids, sucrose, and B) appear to be between 30 and 40 seed m^-2^. Rates beyond 40 seed m^-2^ may result in a negative effect on some seed constituents, possibly due to interplant competition for available nutrients and shade effects. The constituents most affected by management practices, and in our case PD, SR, and RT, appear to be protein, oleic and linolenic acids, sucrose, and B. Since the physiological and biochemical mechanisms of how management practices affect seed constituents are scarce, further research is needed to understand the mechanisms controlling these responses. The increase of stachyose in 2010 may be due to a drier year and high temperature in 2010 compared with 2009, suggesting a possible role of stachyose as an environmental stress compound, and further research is needed to confirm this observation. The current information benefits growers and breeders for considering environmental factors such as heat, drought, and agronomic practices in producing seed with higher quality, especially under double-cropping production systems of soybean and wheat in the Midsouth USA.

## Conflict of Interest Statement

The authors declare that the research was conducted in the absence of any commercial or financial relationships that could be construed as a potential conflict of interest.
